# Dynamics and Microevolution of *Vibrio parahaemolyticus* Populations in Shellfish Farms

**DOI:** 10.1128/mSystems.01161-20

**Published:** 2021-01-12

**Authors:** Songzhe Fu, Qingyao Wang, Yixiang Zhang, Qian Yang, Jingwei Hao, Ying Liu, Bo Pang

**Affiliations:** a College of Marine Science and Environment, Dalian Ocean University, Dalian, China; b Key Laboratory of Environment Controlled Aquaculture (KLECA), Ministry of Education, Dalian, China; c CAS Center for Excellence in Molecular Plant Sciences, Shanghai Institutes for Biological Sciences (SIBS), Chinese Academy of Sciences, Shanghai, China; d University of Chinese Academy of Sciences, Shanghai, China; e Center for Microbial Ecology and Technology, Ghent University, Ghent, Belgium; f State Key Laboratory of Infectious Disease Prevention and Control, National Institute for Communicable Disease Control and Prevention, Chinese Center for Disease Control and Prevention, Beijing, China; University of Hawaii at Manoa

**Keywords:** *Vibrio parahaemolyticus*, multilocus sequence typing, mobile genetic elements, microevolution

## Abstract

Vibrio parahaemolyticus is becoming the leading cause of acute bacterial gastroenteritis, but its population dynamics in aquafarms have received limited attention. To address this research gap, we selected three shellfish farms to examine the impacts of ocean currents and the transport of live aquatic animals on the transmission and microevolution of V. parahaemolyticus by using multilocus sequence typing (MLST) and whole-genome sequencing. MLST and genomic analysis revealed that the community structure of V. parahaemolyticus in Dalian and Donggang was relatively stable in the presence of ocean currents; however, horizontal gene transfer of mobile genetic elements (MGEs) between Dalian and Donggang was very common. Further analysis indicated that the transport of live aquatic animals from Dalian to Xiamen not only introduced new V. parahaemolyticus populations but also allowed the exchange of genetic material between the two sites. More interestingly, Dalian-originated strain ST722 was introduced to Xiamen farms, resulting in one MLST allele change and the acquisition of two genomic islands from indigenous isolates in Xiamen within 8 months; such alterations are thought to promote the adaptation of V. parahaemolyticus. These results provide direct observations of how ocean currents and the transport of live aquatic animals contribute to the dissemination and genetic mixture of V. parahaemolyticus, which provides insights into the dynamics and microevolution of V. parahaemolyticus in aquacultural environments.

**IMPORTANCE** Globally, V. parahaemolyticus-related gastroenteritis outbreaks caused by seafood consumption represent an increasing threat to human health. Despite advances in our understanding of the global epidemiology of pandemic V. parahaemolyticus, fundamental questions about the key driving forces for the spread of V. parahaemolyticus at regional and national scales remain unanswered. This study revealed that the transregional transport of aquatic animals and the movement of ocean currents both contributed to the mixing of V. parahaemolyticus populations. More importantly, this study demonstrated how genetic mixture occurred between introduced and endemic V. parahaemolyticus populations via the transport of aquatic animals, which accelerated bacterial adaptation by transferring ecologically important functions. These results suggest that human activities entail a risk of the emergence of new virulent populations for both aquatic animals and humans by horizontal gene transfer and provide important insights into the microevolution and population mixing of V. parahaemolyticus.

## Abstract (precis)

Globally, V. parahaemolyticus-related gastroenteritis outbreaks caused by seafood consumption represent an increasing threat to human health. Despite advances in our understanding of the global epidemiology of pandemic V. parahaemolyticus, fundamental questions about the key driving forces for the spread of V. parahaemolyticus at regional and national scales remain unanswered.

## INTRODUCTION

Vibrio parahaemolyticus is a Gram-negative bacterium that is widely spread in warm estuarine and marine environments throughout the world (1). This organism has been isolated from a variety of seafood worldwide, including shrimp, fish, and shellfish (2–4). Consumption of raw and undercooked seafood is a major human infection route for pathogenic V. parahaemolyticus (5). In China, V. parahaemolyticus caused more than 100 foodborne outbreaks and 2,000 human infections per year from 2011 to 2016 (6). Knowledge of how V. parahaemolyticus is disseminated in affected areas remains scarce. Multiple transmission modes, including spreading by ocean currents, animal migration, and transportation of aquatic animals or trading of seafood products, might all play roles in the spread of this human pathogen (1). A previous study showed that V. parahaemolyticus could be divided into four diverse populations: VppUS1, VppUS2, VppX, and VppAsia (7). Moreover, the authors found that VppX and VppAsia are distributed worldwide, but the mixing of VppX and VppAsia populations occurred recently. Therefore, it is necessary to identify the key factors that have influenced the recent dissemination of V. parahaemolyticus, which would greatly improve our capacity to predict and mitigate disease occurrence.

Previous studies suggested that both natural and human activities could contribute to the dissemination of V. parahaemolyticus (7). Ocean currents are one of the most widely recognized transmission routes for the spread of V. parahaemolyticus. Ocean currents have often been proposed as a vehicle for the spread of human *Vibrio* diseases through the dispersal of pathogenic specimens attached to zooplankton (8, 9). Recent genomic studies are providing evidence that El Niño may represent a long-distance corridor for waterborne diseases into South America from Asia (10, 11). The emergence of V. parahaemolyticus infections in Alaska, Chile, Peru, and Spain was also associated with the concurrent arrival of warm oceanic waters along the coast (10, 12). However, the above transmission events occurred only during specific climate abnormalities involving ocean currents. Whether ocean currents are a general and major vehicle promoting the global dissemination of V. parahaemolyticus remains unclear.

V. parahaemolyticus can also spread via the migration of animals. Previous studies found that bird-carried *Vibrio* strains could be acquired through the direct predation of local mollusks (13). Transmission of other waterborne pathogens assisted by fish movement has also been confirmed (14). However, due to the low prevalence of V. parahaemolyticus in wild animals (13), long-distance natural movement of waterbirds and other aquatic organisms may not necessarily provide habitats suitable for the persistence of V. parahaemolyticus and thus may not be sufficient to alter endemic V. parahaemolyticus populations.

Human activities, however, may be another driving force to promote the rapid dissemination of V. parahaemolyticus populations via the deliberate transport of aquatic animals to facilitate aquaculture and subsequent sales in seafood markets. Analysis of zooplankton and seawater by Martinez-Urtaza et al. showed that the occurrence of V. parahaemolyticus in offshore areas was almost exclusively associated with zooplankton abundance (12). Since V. parahaemolyticus tends to attach to plankton to resist adverse environments and to obtain nutrients, it is likely to travel with plankton into shellfish farms by ocean currents (15). However, shellfish also tend to concentrate V. parahaemolyticus within their tissues by using plankton as food sources and therefore also represent an important ecological niche for this bacterium (4). Thus, high-density shellfish farming activities may create a melting pot for V. parahaemolyticus and become an important source for pathogens. However, this fact has not been confirmed epidemiologically. The extent to which ocean currents and human activities contribute to the dissemination of V. parahaemolyticus is still unclear.

Additionally, such dissemination promotes the microevolution of V. parahaemolyticus. Loyola et al. have reported that single-nucleotide variants (SNVs) and horizontal gene transfer (HGT) both contribute to genome diversification of V. parahaemolyticus in oceanic environments; the extent of the effects of these evolutionary forces on individual isolates likely differs and depends on the ecological scenario of each isolate (16). Due to the extensive use of antibiotics, HGT of antibiotic resistance by integrative and conjugative elements (ICEs) has become another driving force shaping the genome plasticity of V. parahaemolyticus in aquacultural environments (17). The high levels of particulate organic matter in the farming region also result in the high abundance of zooplankton in shellfish farms, which not only promotes the growth and population mixing of V. parahaemolyticus but also drives HGT and environmental adaptation (18). Therefore, it is important to study both natural and human factors in shellfish farms to better understand the links between farming activities and the evolution of V. parahaemolyticus populations and genomes.

The continuous surveillance of the dynamics and microevolution of V. parahaemolyticus populations in aquaculture regions has been a longstanding issue. It remains unclear to what extent (i) the dynamics of V. parahaemolyticus populations in shellfish farms and (ii) the ocean currents and transport of live aquatic animals contribute to the expansion and genetic mixing of V. parahaemolyticus.

In numerous studies, multilocus sequence typing (MLST) has been used to describe the population structures of V. parahaemolyticus. For instance, Han et al. presented a population structure analysis of 490 V. parahaemolyticus isolates from clinical samples in 17 coastal countries using MLST, which divided the 161 clinically relevant sequence types (STs) into eight clonal complexes (19). Continuous surveillance of V. parahaemolyticus in aquafarms with the exchange of live aquatic animals or ocean current offers an opportunity to observe such evolutionary changes, which are often obscured by the dynamics of V. parahaemolyticus populations.

**FIG 1:**
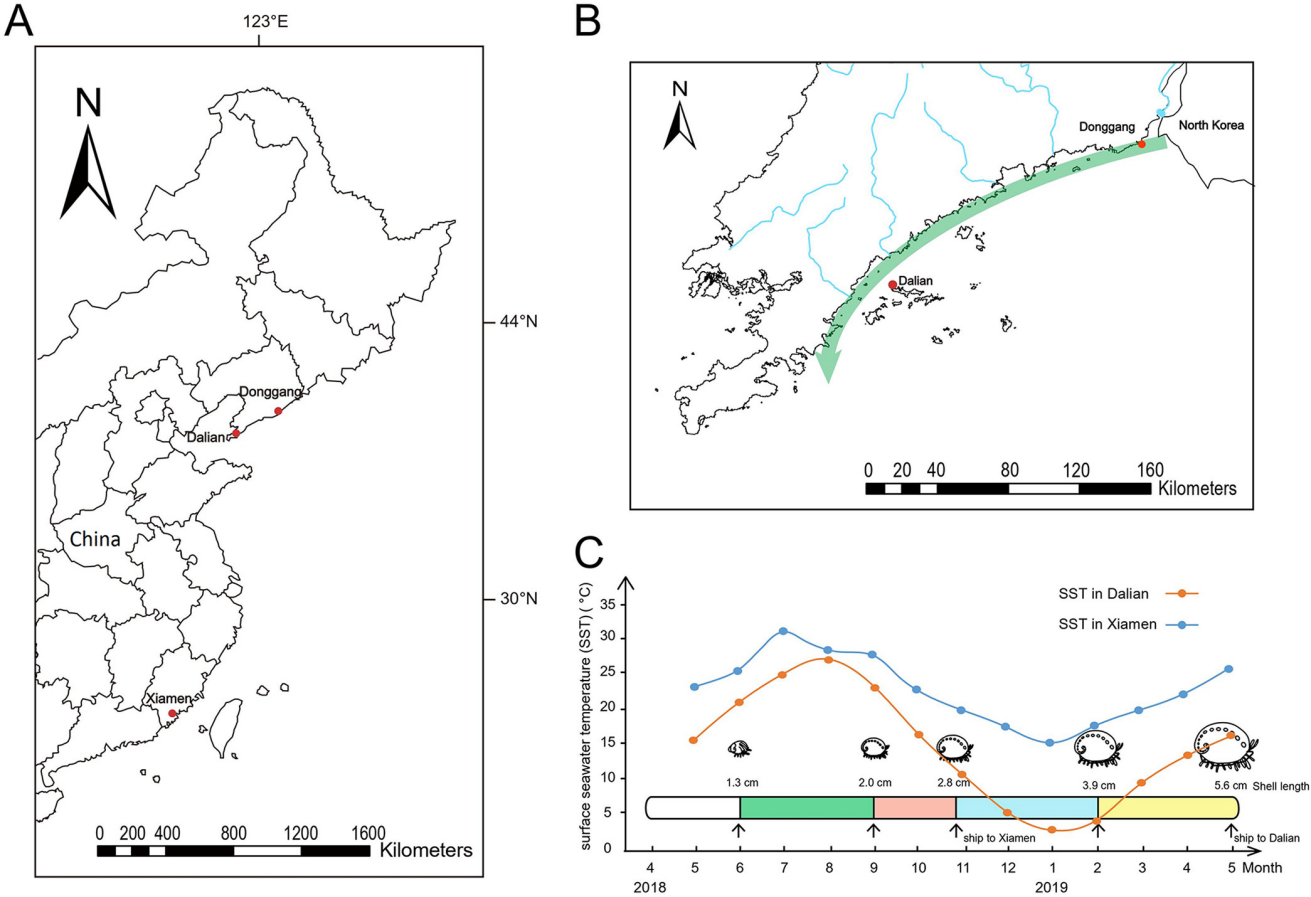
Sampling sites and research design. (A) The locations of three selected shellfish farms. (B) Sampling sites along the ocean current in South Liaodong peninsula. The green arrow indicates the direction of the ocean current. The blue lines indicate rivers. (C) The north-south transfer of the abalones from Dalian to Xiamen. The *y* axis indicates the seawater temperature; the *x* axis indicates the study period from April 2018 to May 2019. Black arrows indicate the sampling times. The maps were created using ArcGIS Desktop 10.2 software.

Here, we conducted a 1-year investigation to evaluate the effects of aquatic animal exchange and ocean currents on the presence and temporal trend of V. parahaemolyticus populations in seawater by MLST. Shellfish farms at three sites were selected for pairwise comparisons (Fig. 1A), including two shellfish farms in Dalian and Donggang located along the ocean current (Fig. 1B). To understand the impacts of the transfer of aquatic animals between two farming regions on the local community composition of V. parahaemolyticus populations, we sampled populations in two shellfish farms in Dalian and Xiamen, in which juvenile abalones from Dalian were transported to Xiamen with warmer seawater temperature during winter (Fig. 1C). Additionally, we used whole-genome sequencing to characterize the genetic mixing during the dissemination of V. parahaemolyticus, presenting an integrated view of the timeline of population mixing for this species.

## RESULTS

### Transmission of V. parahaemolyticus populations by ocean currents.

To elucidate whether ocean currents are the main driver behind the population mixing of V. parahaemolyticus, we selected two sites (shellfish farms in Dalian and Donggang) that are affected by an alongshore current and sampled the seawater in June 2018, September 2018, October 2018, February 2019, and May 2019 (Fig. 1B). The abundance of V. parahaemolyticus was measured by the colony formation unit (CFU) assay.

In Dalian, 92, 108, 135, 62, and 205 isolates of V. parahaemolyticus were obtained from 6 liters of seawater in June 2018 (15.3 CFU/liter), September 2018 (18 CFU/liter), October 2018 (22.5 CFU/liter), February 2019 (10.3 CFU/liter), and May 2019 (34.2 CFU/liter), which could be subtyped into 17, 21, 20, 15, and 25 sequence types (STs), respectively. Good’s coverage value of each sampling time ranged from 88.0% to 90.5%, indicating a high representativeness of the V. parahaemolyticus population structure (Table 1). The Shannon index and Simpson index in Dalian ranged from 2.23 to 2.66 and from 88.7 to 91.7, respectively. In total, 12 STs were found at all sampling time points (Fig. 1A). ST658 (average proportion 19.91%), ST114 (14.63%), ST180 (14.13%), ST693 (14.13%), and ST1743 (9.78%) were the top 5 STs dominating the V. parahaemolyticus communities in Dalian. Notably, ST3, a predominant ST in clinical samples, accounted for only 2.17% to 6.34% of STs in the oceanic environments of Dalian, which is consistent with its low prevalence reported previously (10). Likewise, the occurrences of another *trh*-positive genotype, ST1750, were low, accounting for 2.74% to 5.91% throughout the investigated period.

**TABLE 1:** Diversity indices and richness index of each sampling site^*a*^

Sample ID	No. of STs	No. of clones	No. of singleton STs	Good’s coverage (%)	Simpson diversity	Shannon diversity
DL-J	17	92	1	94.1	87.1	2.23
DL-S	21	109	2	90.5	91.1	2.65
DL-O	20	135	2	90.0	91.7	2.63
DL-F	15	62	1	80.0	88.7	2.41
DL-M	25	205	3	88.0	90.6	2.66
DH-J	12	69	2	83.3	86.3	2.20
DH-S	14	83	3	78.6	90.5	2.45
DH-O	15	75	4	73.3	89.8	2.42
DH-F	10	37	1	90.0	86.9	2.14
DH-M	16	120	3	81.3	91.0	2.51
XM-J	19	129	2	89.5	90.2	2.65
XM-S	26	204	3	88.5	91.2	2.71
XM-O	24	199	3	87.5	90.8	2.67
XM-F	18	75	2	88.8	89.7	2.55
XM-M	30	410	1	96.6	91.6	2.85

a Shannon index (H = −∑Pi × ln Pi), Simpson index (D = 1 − ∑Pi^2^); C = 1 − n1/N. n1 is the number of singletons, while N is the total number of STs. DL-J, DL-S, DL-O, DL-F, and DL-M denote Dalian samples collected in June 2018, September 2018, October 2018, February 2019, and May 2019, respectively. DH-J, DH-S, DH-O, DH-F, and DH-M denote samples collected from Donggang in June 2018, September 2018, October 2018, February 2019, and May 2019, respectively. XM-J, XM-S, XM-O, XM-F, and XM-M denote Xiamen samples collected in June 2018, September 2018, October 2018, February 2019, and May 2019, respectively.

**FIG 2:**
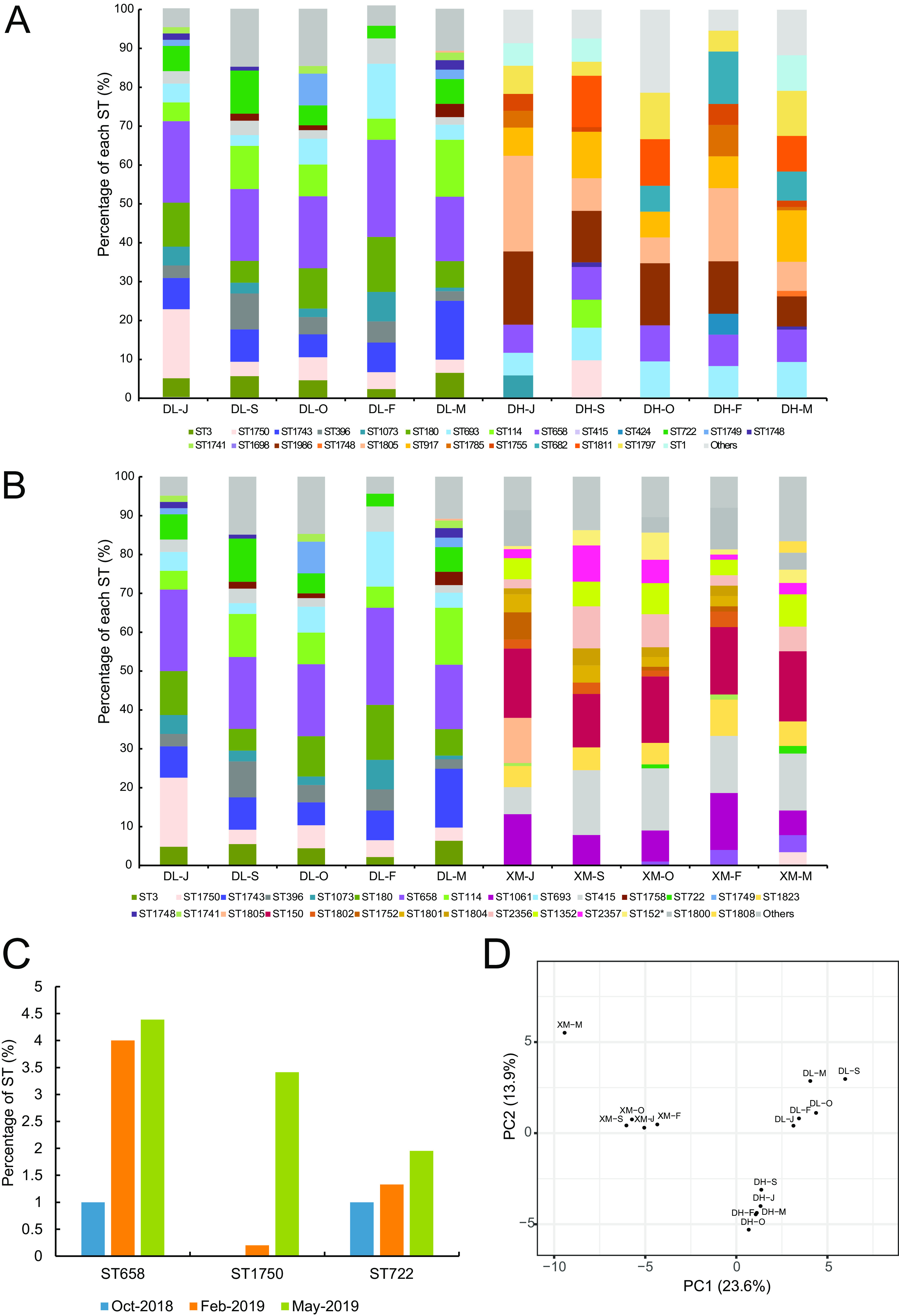
Dissemination of V. parahaemolyticus by the movement of ocean current. (A) Temporal distribution of sequence types (STs) in Dalian and Donggang from June 2018 to May 2019. (B) Dynamics of the STs of V. parahaemolyticus in Dalian and Xiamen from June 2018 to May 2019. (C) The dynamics of three introduced STs of V. parahaemolyticus in Xiamen. (D) Principal-coordinate analysis (PCoA) based on Bray-Curtis dissimilarity of V. parahaemolyticus community composition of different samples from three sites based on the composition of STs. DL-J, DL-S, DL-O, DL-F, and DL-M denote Dalian samples collected in June 2018, September 2018, October 2018, February 2019, and May 2019, respectively; DH-J, DH-S, DH-O, DH-F, and DH-M denote samples collected from Donggang (estuary of the Yalu River) in in June 2018, September 2018, October 2018, February 2019, and May 2019, respectively; XM-J, XM-S, XM-O, XM-F, and XM-M denote Xiamen samples collected in in June 2018, September 2018, October 2018, February 2019, and May 2019, respectively.

A total of 69, 83, 76, 37, and 120 isolates of V. parahaemolyticus were obtained from 6 liters of seawater in June 2018 (11.5 CFU/liter), September 2018 (13.8 CFU/liter), October 2018 (11.5 CFU/liter), February 2019 (6.17 CFU/liter), and May 2019 (20 CFU/liter) in Donggang (the estuary of the Yalu River), which could be subtyped into 12, 14, 15, 10, and 16 STs, respectively. The Shannon index and Simpson index in Donggang ranged from 2.14 to 2.51 and from 89.8 to 91.0, respectively. However, Good’s coverage value ranged from only 73.3% to 90.0%, indicating that some V. parahaemolyticus strains might not be represented in the samples. ST917, ST1805, ST658, ST693, and ST1811 were the most abundant STs in Donggang, and their average relative abundances were 11.29%, 10.38%, 9.79%, 8.74%, and 8.60%, respectively (Fig. 2A). Notably, 10 STs were shared in both Dalian and Donggang, which constituted 54.9% and 45.2% of the total population, respectively. Interestingly, ST1805, which was abundant in all seasons in Donggang, was identified only in May 2019 in Dalian (accounting for 1.95%), indicating a possible introduction of a new population from Donggang to Dalian.

### Population dynamics and dissemination of V. parahaemolyticus by the transportation of live abalone from North to South China.

To examine whether North-South transfer of live abalones would introduce the V. parahaemolyticus into the local environments and accelerate the genetic exchange between V. parahaemolyticus populations, we sampled the seawater in the abalone farm in Xiamen before and after the transfer of live abalones using the method described above.

MLST was conducted for all V. parahaemolyticus isolates at five time points to capture the dynamics of V. parahaemolyticus populations before and after the abalone transfer. The number of V. parahaemolyticus isolates was relatively high in the seawater of Xiamen; 129, 204, 199, 75, and 410 isolates of V. parahaemolyticus were retrieved in June 2018 (21.5 CFU/liter), September 2018 (34 CFU/liter), October 2018 (33.2 CFU/liter), February 2019 (12.5 CFU/liter), and May 2019 (68.3 CFU/liter), respectively. Good’s coverage value ranged from 87.5% to 96.6% in Xiamen samples, indicating high representativeness of the local communities (Table 1). The Shannon indices ranged from 2.55 to 2.85, which were higher than the values calculated from the Dalian and Donggang samples. V. parahaemolyticus communities in Xiamen were dominated by distinct STs (Fig. 2B). In September 2018 and October 2018, seawater samples mainly consisted of ST150 (16.76%), ST415 (14.1%), ST1061 (9.87%), ST2356 (7.03%), and ST1352 (6.66%). Only ST415 was found in both Dalian (accounting for only 3.5%) and Xiamen at all sampling time points. On 31 October 2018, 5 days after receiving the abalones from Dalian, the overall community structure of V. parahaemolyticus remained stable, but ST658 (1%) and ST722 (1%), which were found only in Dalian, were also identified in the seawater of Xiamen (Fig. 2C). The proportions of ST658 and ST722 gradually increased to 2.67% and 1.33% in February 2019, respectively. In May 2019, ST658, ST1750, and ST722 were also identified in the samples, and their relative abundances reached 4.39%, 3.41%, and 1.95%, respectively. Another interesting finding was that ST1799, which differed at only one locus from ST722, emerged in May 2019 in Xiamen.

We also compared the effects of transport of live aquatic animals and ocean currents on the community composition of V. parahaemolyticus by principal-coordinate analysis (PCoA). The community composition of V. parahaemolyticus displayed spatial heterogeneity, with clustering according to sampling areas (analysis of similarities [ANOSIM], *P* < 0.001), but no significant seasonal shifts were observed in Dalian or Donggang samples. The introduction of new populations by the transport of live aquatic animals markedly affected the community compositions, as the Xiamen sample in May was distinct from the remaining four samples (ANOSIM, *P* < 0.001), while five samples from Dalian and Donggang were separately grouped together (Fig. 2D). In addition, community compositions of V. parahaemolyticus in Donggang were also closer to the groups in Dalian.

### Phylogenomic relationship of V. parahaemolyticus.

**FIG 3:**
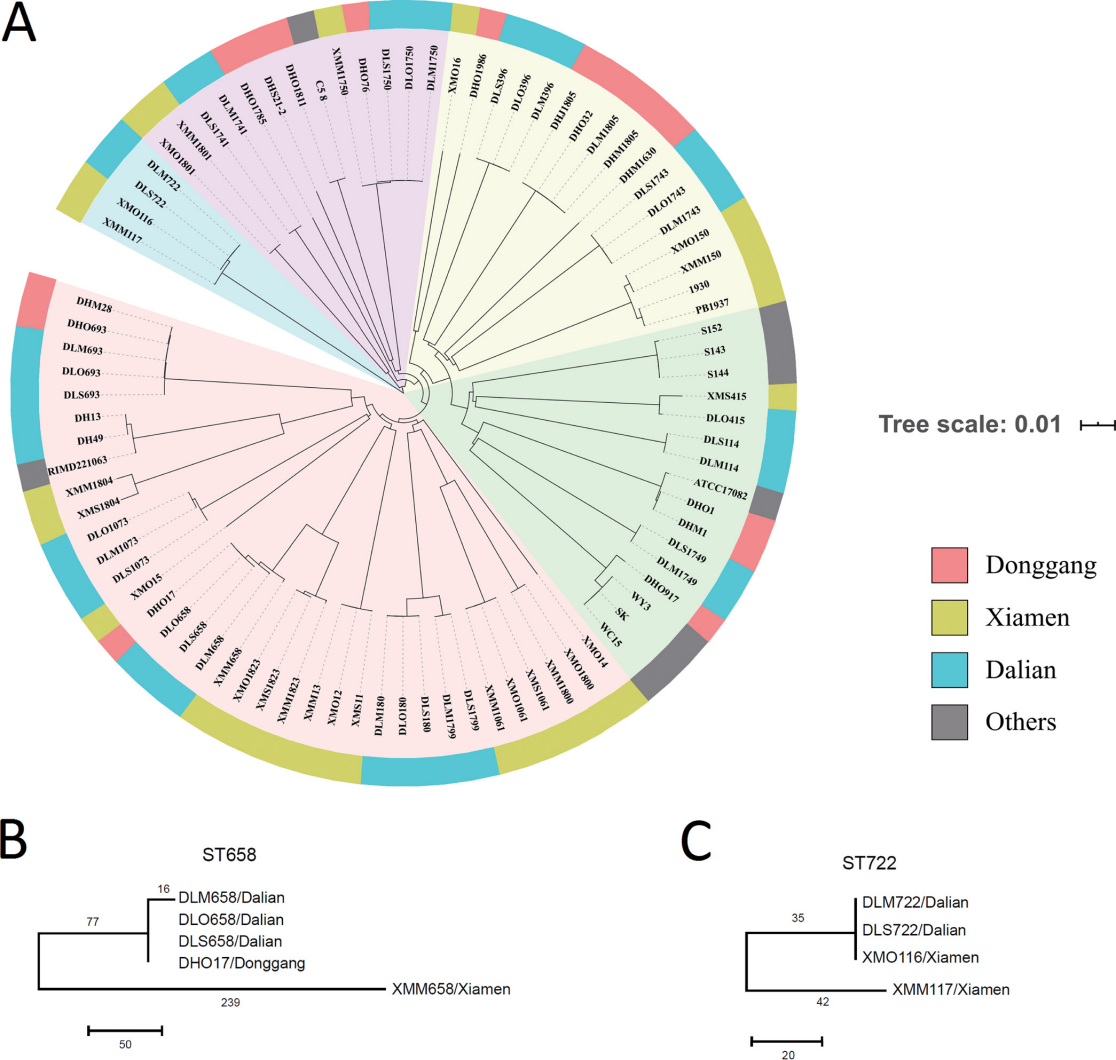
Phylogeny and metadata summary of V. parahaemolyticus genomes obtained from Dalian, Donggang, Xiamen, Shandong province, South Korea, and Japan. (A) Maximum likelihood phylogeny of 85 V. parahaemolyticus genomes from Dalian, Donggang, Xiamen, Shandong province, South Korea, and Japan. Local lineages present in Dalian, Donggang. and Xiamen are highlighted in blue, red, and chartreuse, respectively. The genomes from Shandong province, South Korea, and Japan are indicated in gray. The scale bar denotes substitutions per variable site. (B and C) Geophylogeny of ST658 (B) and ST722 and ST1808 (C) V. parahaemolyticus genomes obtained from Dalian, Donggang, and Xiamen. SNP number is indicated on the branch. The scale bar indicates the number of SNPs.

The STs in each sampling site accounting for over 2% of the total isolates were selected for whole-genome sequencing (see Table S1 in the supplemental material). In a previous study, genomes with fewer than 10 single nucleotide polymorphism (SNP) differences were defined as genomically related clones, while strains with a pairwise mutational core genome SNP difference of fewer than 2,000 SNPs were categorized into the same population (20). We adopted these criteria for justifying the relationship of V. parahaemolyticus populations. Phylogenomic analysis was performed and showed that the isolates from three farms were distributed in five clusters of the phylogenetic tree; most of the strains within the same STs were tightly clustered together with 0 to 10 SNPs except for ST1804 and ST658 (Fig. 3A). Genomic analysis also confirmed that ST1805, ST693, ST658, and ST1805 isolates from Donggang were all genomically related to those found in Dalian (Fig. 3A).

10.1128/mSystems.01161-20.3 TABLE S1 List of strains sequenced in this study and their plasmid profile. Download Table S1, XLSX file, 0.02 MB. Copyright © 2021 Fu et al. 2021 Fu et al. This content is distributed under the terms of the Creative Commons Attribution 4.0 International license.

To confirm the fine-resolution epidemiological relationships of STs found in both Xiamen and Dalian, we further calculated pairwise SNP differences based on the core genome SNPs for 7 STs (ST1750, ST415, ST180, ST658, ST722, ST1808, and ST1799) separately. These STs contained isolates retrieved at all five time points from Dalian and Xiamen as well as isolates from other regions or countries. The results showed that the ST1750 strains isolated from Dalian were genomically identical to that from Xiamen with one SNP difference. The ST658 strain from Xiamen had 316 SNP differences relative to the Dalian strains (Fig. 3B). ST415 strains were found at both sites. Compared to the ST415 Dalian strains, the ST415 Xiamen strains differed by 2,206 SNPs, indicating an earlier divergence between the two sites. In Dalian, the ST1799 strain, with a one-locus difference from ST180, had 7,224 SNP differences from ST180 strains. In contrast, the ST722 and ST1808 strains, which also have a one-locus difference, had only 77 SNP differences (Fig. 3C).

### Microevolution of V. parahaemolyticus strains differing by one MLST locus.

**FIG 4:**
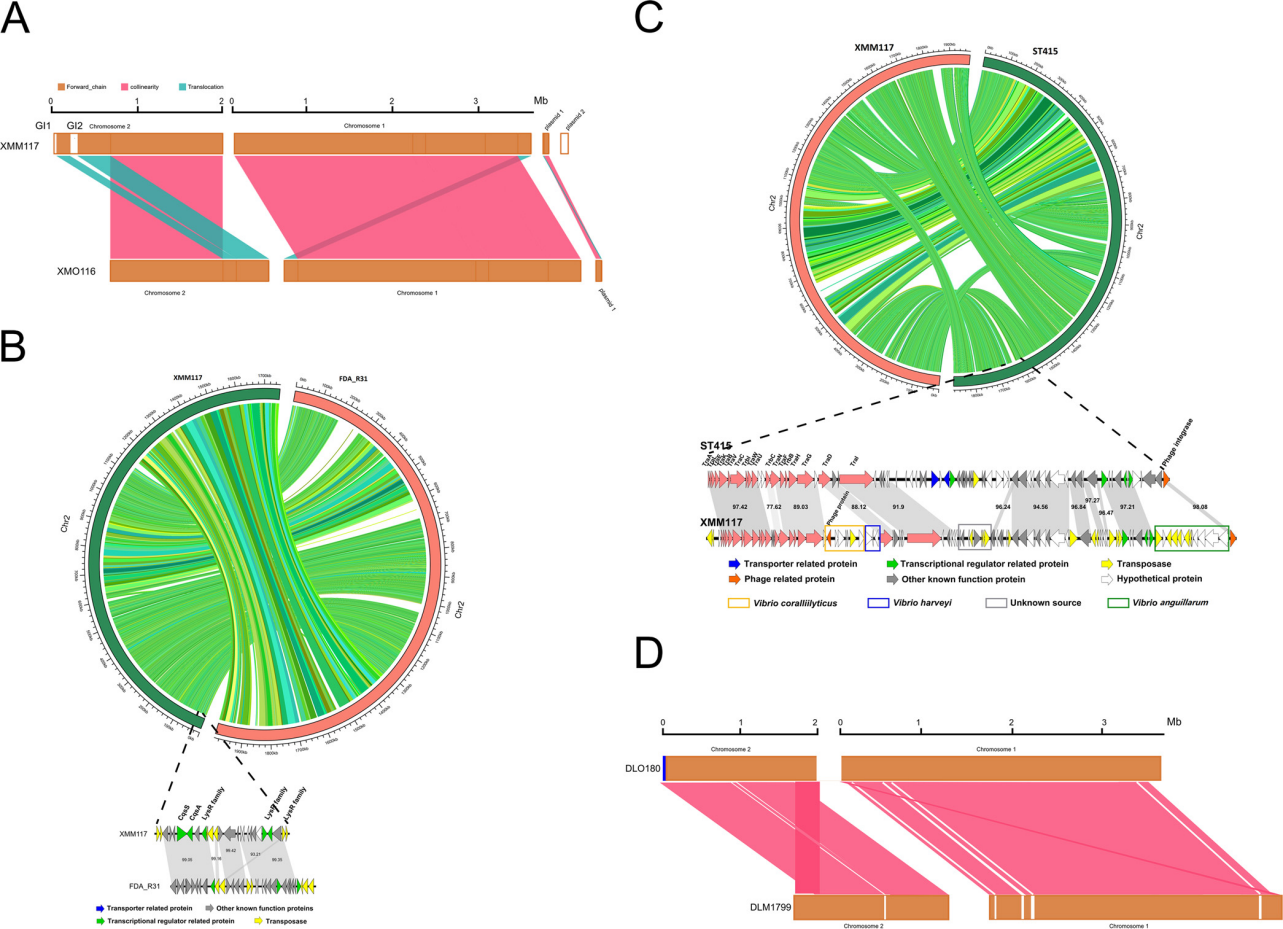
Comparative genome analysis between the V. parahaemolyticus strains XMO116 and XMM117 and between strains DLM1799 and DLO180. (A) Linear comparison between genomes of XMO116 and XMM117. Inverted regions in XMO116 are clearly depicted as blocks below a genome’s center line. Two genomic islands, namely, GI1 and GI2, are indicated in chromosome 2. (B) Linear comparison of chromosome 2 between genomes of XMM117 and FDA_R31 showing the schematic representation of genomic island 1 (GI1) and its homology to U.S. strain FDA_R31. (C) Linear comparison of chromosome 2 between genomes of XMM117 and ST415 strain XMO415 showing the schematic representation of genomic island 2 (GI2) and its homology to the ST415 strain. (D) Linear comparison of genomes of DLM1799 and DLO180.

Subsequently, we looked for the signatures of microevolutions (including intrinsic mutations and genetic recombination) between the ST722 strain XMO116 and the ST1808 strain XMM117, which differed by only one locus based on the MLST typing scheme. Comparative genomic analysis revealed that XMM117 had two additional genomic islands and one additional plasmid, pXMM117-2 (Fig. S1), that were absent in strain XMO116 (Fig. 4A). The first genomic island was a 28.5-kb region flanked by two transposases and contained genes encoding a transcriptional regulator (LysR family), an aconitate hydratase, the CqsA-CqsS system, and LeuO (a transcriptional activator of the *leuABCD* operon) (Fig. 4B). This segment had over 99% identity with the corresponding region in two U.S. strains, FDAARGOS_667 and FDA_R31. In addition, this segment also harbored several insertion sequences (ISs), which further divided it into three small genomic islands.

10.1128/mSystems.01161-20.1 FIG S1 Diagrams of the plasmids found in this study generated by DNAPlotter. Open reading frames (ORFs) were predicted by RAST; the innermost circle shows a GC plot, with dark yellow representing below-average GC content and brown indicating above-average GC content. Download FIG S1, PDF file, 2.0 MB. Copyright © 2021 Fu et al. 2021 Fu et al. This content is distributed under the terms of the Creative Commons Attribution 4.0 International license.

The second genomic island, located between positions 188185 and 277598 in chromosome 2 of strain XMM117, harbored an IncF plasmid-like conjugative transfer cassette and six phage-like genes, the majority of which likely came from an ST415 V. parahaemolyticus strain, XMO415 (Fig. 4C). However, there were four segments that were not present in the ST415 strains but had diverse origins, and these segments might have originated from Vibrio coralliilyticus, Vibrio harveyi, Vibrio anguillarum, and an unknown source. Notably, compared with the ST415 strains, 17 transposases were identified in the genomic island, indicating that transposases might play vital roles in the acquisition of new genetic elements. The origins of the genes with hypothetical functions are unknown.

Apart from the acquisition of external genetic materials, SNPs were also found in strain XMM117. Compared with strain XMO116, 47 nonsynonymous and 30 synonymous SNPs were identified in strain XMM117, and one nonsynonymous mutation resulted in the change of aspartic acid to l-asparagine (Table S2). In addition, amino acid changes were also identified in several functional proteins such as serine hydroxymethyltransferase and DNA gyrase subunit A. Also, one stop mutation occurred in a gene encoding an uncharacterized protein on chromosome 2.

10.1128/mSystems.01161-20.4 TABLE S2 Nature of SNPs in strain XMM117 relative to strain XMO116. Download Table S2, XLSX file, 0.02 MB. Copyright © 2021 Fu et al. 2021 Fu et al. This content is distributed under the terms of the Creative Commons Attribution 4.0 International license.

In contrast, large-scale import of genetic material was not observed between ST180 and ST1799 strains. Compared with ST180, only five small genomic islands (3 to 10 kb) were lost in ST1799 strains (Fig. 4D). ST1799 strain DLS1799 has a 7,224-SNP difference relative to ST180 strain DLO180 in 678 genes with 1,115 nonsynonymous and 6,092 synonymous changes, the majority of which (629 genes) have only or have more synonymous changes, indicating that ST1799 may undergo a purifying selection (Table S3). In addition, early stop mutation events were also found in 17 genes.

10.1128/mSystems.01161-20.5 TABLE S3 Nature of SNPs in strain DLM1799 relative to strain DLO180. Download Table S3, XLSX file, 0.4 MB. Copyright © 2021 Fu et al. 2021 Fu et al. This content is distributed under the terms of the Creative Commons Attribution 4.0 International license.

Next, we compared the biofilm formation and the expression of *cpsA* between ST722 and ST1808 strains. Biofilm formation assays showed that the biomass of biofilms in strain XMM117 was significantly greater than that of another two ST722 strains (*P* < 0.01, Fig. S2A). Likewise, quantitative reverse transcription-PCR (qPCR) assay showed that strain XMM117 exhibited 3.2-fold-higher expression of *cpsA* than the ST722 strains (*P* < 0.01) (Fig. S2B), suggesting that the acquisition of the genomic island encoding the CqsA-CqsS system and LeuO might improve the environmental adaptation of V. parahaemolyticus (20).

10.1128/mSystems.01161-20.2 FIG S2 Phenotype assays of three V. parahaemolyticus strains. (A) The biofilm formation assay; the biomass of biofilm was measured by MTT at OD_550_. Error bars indicate the standard deviation from three replicate cultures. (B) Relative expressions of *cpsA* in different strains. The expression of *cpsA* in strain ATCC 17802 was set at 1, and the expression in other strains was normalized accordingly using the 2^−ΔΔ^*^CT^* method. **, significant differences (*P* < 0.01). Download FIG S2, PDF file, 0.1 MB. Copyright © 2021 Fu et al. 2021 Fu et al. This content is distributed under the terms of the Creative Commons Attribution 4.0 International license.

### Horizontal gene transfer (HGT) of plasmid and mobile genetic elements (MGEs).

The discovery of a genomic island originating from other genotypes in an ST1808 strain, XMM117, prompted us to investigate the transfer of the mobilome between different sites.

First, we compared the plasmid profiles among isolates from Dalian, Donggang, and Xiamen. The results showed that plasmids were abundant in the oceanic environments of Dalian and Donggang, among which eight distinct plasmids were identified in 34 out of 48 Dalian and Donggang isolates (70.8%). Three types of plasmids were shared between different STs. In contrast, only one plasmid associated with shrimp disease was identified in two out of 32 Xiamen isolates before October 2018. However, the transportation of aquatic animals not only introduced new STs but also allowed the HGT of plasmids. One Dalian plasmid, pDLS722-1 from ST722, was also identified in ST150 isolates from Xiamen, indicating the horizontal transfer of the plasmid along with the spread of novel populations. Likewise, the plasmid pTetB-VA1 encoding tetracycline resistance was found in both ST1750 and ST150. Notably, p1937-2 was found in both ST1750 strains from Dalian and in ST150 strains from Xiamen, as well as the strains isolated from Shanghai and Zhejiang (21), indicating that p1937-2 was widely distributed in the coastal region, probably due to the transport of live animals. Overall, plasmids were abundant in Dalian and Donggang isolates, and the transport of aquatic animals might facilitate plasmid transfer from regions of high plasmid abundance to sites with low plasmid prevalence.

**FIG 5:**
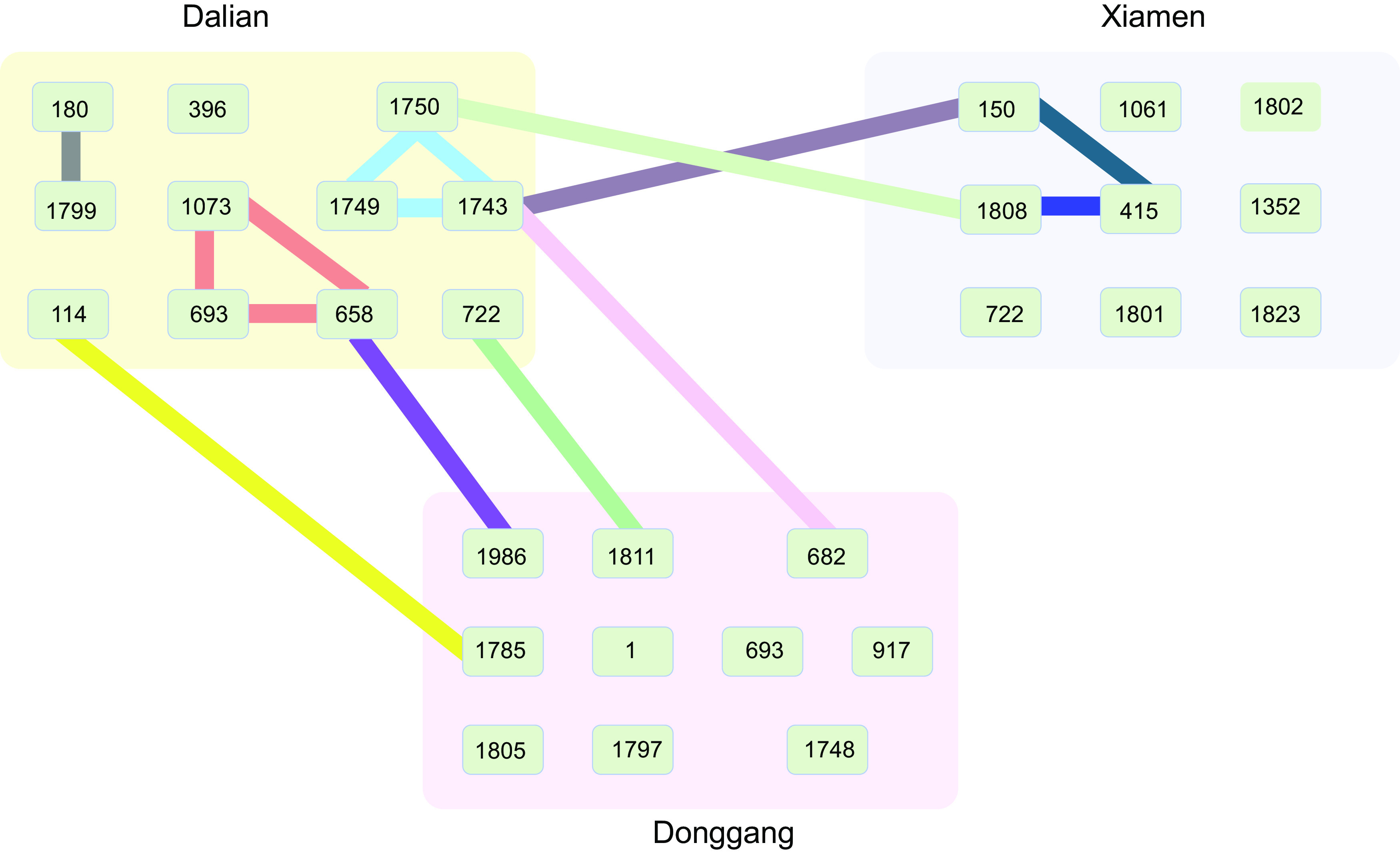
Horizontal gene transfer (HGT) of plasmid and mobile genetic elements (MGEs) between Dalian, Donggang, and Xiamen V. parahaemolyticus populations. The predominant STs in each site were labeled in each ellipse. Blackish green line, HGT of plasmid pVA1; blue line, HGT of MGE; aquamarine line, HGT of pTetB-VA1; caramel line, HGT of prophage; light blue line, HGT of plasmid p1937-2; red line, HGT of pVPSD2016-1; pink line, HGT of MGE containing toxin-antitoxin system; yellow line, HGT of plasmid pVOWZ1; green line, HGT of MGE containing CqsA/CqsS; purple line, HGT of MGE containing CcdA-CcdB toxin-antitoxin system; gray line, HGT of MGE containing RNA-directed DNA polymerase.

“Genome island-like” regions are the most common type of MGEs. We found 52 such regions flanked by two transposases with potential transferability in the isolates from Dalian, Donggang, and Xiamen (Table S4). Interestingly, in the isolates from Dalian and Donggang, the genomes were rich in MGEs, among which 11 MGEs came from other species. For instance, the genomes of ST658, ST1743, and ST722 harbored 9, 5, and 3 MGEs, respectively, from other STs or other species (Table S4). The HGT of MGEs between Dalian and Xiamen was also very common (Fig. 5). For example, a prophage (MGE27) containing an RstA phage-related replication protein, a bacteriophage f237 repressor, zona occludens toxins (*zot*), an accessory cholera toxin (*ace*), and seven hypothetical proteins was found in the plasmid and chromosome of three STs, suggesting that it might be a virulent MGE (Table S4). Similarly, a genomic island containing *cqsA* and *cqsS* was found in both ST1811 and ST722. In contrast, MGEs were rarely found in the genomes of strains from Xiamen; only ST1061 and ST1800 presented three MGEs. HGT occurred only between ST415 and ST150, and the subsequent transport of aquatic animals might have allowed the transfer of two genomic islands from ST415 and ST23 into ST1808. Other HGTs from Xiamen isolates to ST658 and ST1750 were not observed, probably because the observation period was short.

10.1128/mSystems.01161-20.6 TABLE S4 Mobile genetic elements (MGEs) found in sequenced strains in this study. Download Table S4, XLSX file, 0.1 MB. Copyright © 2021 Fu et al. 2021 Fu et al. This content is distributed under the terms of the Creative Commons Attribution 4.0 International license.

## DISCUSSION

A previous study suggested that mixing of V. parahaemolyticus populations recently occurred (7). However, most investigated strains were collected from seafood or clinical samples and thus do not represent a defined epidemiological cohort. Here, we conducted a 1-year investigation directed toward evaluating the effects of aquatic animal exchange and ocean current on the presence and temporal trend of V. parahaemolyticus populations in aquaculture environment by multilocus sequence typing (MLST). To explain the dispersal pattern, we introduced concepts of bacterial communities to describe the intraspecies diversity and dynamics of V. parahaemolyticus. Here, ST was used as an equivalent to operational taxonomic units (OTUs) to describe V. parahaemolyticus populations, and we introduced three microbial diversity indices to describe the diversity of V. parahaemolyticus. Using these concepts, we examined the impacts of ocean currents and the transport of live aquatic animals on the population mixing of V. parahaemolyticus. The results here clearly showed that these two transmission routes greatly impact the population mixing of V. parahaemolyticus on different time scales.

The transport of live aquatic animals is a pronounced route for introduction of STs into aquaculture farms, which can be observed over a few months. Notably, some STs such as ST658 accounted for up to 4.59% of the total community, indicating that a few of the introduced V. parahaemolyticus populations had been established and fitted well in their new environment. This finding provides evidence supporting the hypothesis proposed by Yang et al. that V. parahaemolyticus can multiply rapidly to constitute a substantial proportion of the bacteria in its new habitat (7). Thus, the transport of live aquatic animals deliberately delivered to distant farms in large quantities could have facilitated the transmission of bacteria among different regions or countries. For our cases, the epidemiological investigation showed that a large number of abalone, sea cucumber, and other aquatic animals were transported from Liaoning and Shandong to Fujian province from later October to early November, as high temperatures in South China would promote the growth of aquatic animals and shorten their life cycle (22). Such large-scale movement of live aquatic animals from north to south (or the reverse direction) would have profound effects on the mixing of V. parahaemolyticus populations.

To examine whether ocean current also contributes to the dissemination of V. parahaemolyticus, we selected two sites in the start and end of an ocean current along the Liaodong peninsula. The seasonal variation of the ocean current mainly depends on the runoff of the Yalu River and the wind in southern Liaoning, which provided an exceptional example to examine whether ocean current promotes the dissemination of V. parahaemolyticus. Not surprisingly, the community structures in Donggang and Dalian were similar as 12 STs were shared in the two sites. Our previous surveillance showed that ST1805 has been frequently isolated in a bivalve farm in Donggang since 2017 (data not shown). However, ST1805 was identified only in May 2019 in Dalian (accounting for only 0.48%), indicating that the spread of V. parahaemolyticus between two sites would possibly take years. As the ocean current selected in our study lasts for only <200 km, it is reasonable to speculate that the spread of bacteria between the oceans would be further limited by large distances as bacteria might have less chance of survival between them.

Moreover, our study also revealed that the annual introduction of shellfish from north to south (or vice versa) for aquaculture may also greatly promote the genetic exchange of V. parahaemolyticus populations. Population genetic theory implies that once a migrant population arrives in a new deme, it will progressively import DNA from other strains and become increasingly similar to the other strains (23). Our results also supported this theory. MGEs and plasmids enable the frequent recombination of genetic materials in both Dalian and Donggang, which may facilitate the environmental adaptation of V. parahaemolyticus. Our study also showed that transportation of aquatic animals from Dalian to Xiamen enabled the rapid recombination of genetic elements in strain XMM117. Such transportation not only introduces new populations but may also promote bacterial evolution and lead to genome plasticity, which is frequently accomplished by the acquisition of new genes (18). For instance, strain XMM176, which originated from Dalian, progressively imported DNA from other V. parahaemolyticus strains (including the genetic segment found in U.S. strains). These observations suggested that genetic exchange frequently occurs when the strains enter a new environment. Due to the wide use of antibiotics in abalone aquaculture, such large-scale seafood transportation can greatly accelerate the mixing of V. parahaemolyticus populations and also enable the transfer of MGEs with antibiotic resistance genes (17).

Interestingly, the abundance of MGEs in Dalian and Xiamen is remarkably different, which might be associated with the location and aquaculture model. The Dalian shellfish farm is located in open seawater, which is subject to the currents from at least three directions. Likewise, V. parahaemolyticus populations in Donggang are also largely affected by runoff from the Yalu River. Thus, both sites created a melting pot to facilitate genetic exchange among different populations. In contrast, recirculated seawater was used in the indoor abalone farm in Xiamen with only 10% water exchange weekly. Future research is needed to confirm whether this aquaculture model can reduce microevolution and HGT within V. parahaemolyticus populations.

A limitation of our study is that the surveillance period was relatively short and thus might not reflect the long-term effects of human activities on the dissemination of V. parahaemolyticus. Additionally, whether ST can be used as a subtype unit to describe the population dynamics remains unknown. For instance, in this study, ST415 was divided into two populations. Nevertheless, the observation of rapid dissemination of pathogens is informative for population migration patterns. These results suggested that the dissemination and recombination of V. parahaemolyticus populations among aquatic animals occurred rapidly. Another limitation is that there is no replication of farms connected by ocean current or of live shellfish transport. However, there are several other locations that would be suitable for further studies. For instance, there are abundant shellfish farms along the Shandong peninsula; the effects of ocean currents between the Bohai Sea and the Huanghai Sea on the population structures of V. parahaemolyticus are worthy of investigation. Additionally, the impacts of seasonal ocean wind on the dissemination of *Vibrio* sp. are worth exploring. For instance, a previous study showed that the dissemination of V. cholerae O139 completely corresponds to the direction of seasonal Indian Ocean wind (from the east to the northwest) (24). In the future, more coastal shellfish farms along ocean currents or shellfish farms with an annual transfer of shellfish will be selected to monitor the dynamics of V. parahaemolyticus populations. Ultimately, the low Good’s coverage value (below 85%) of some sampling times might not fully reflect the genetic diversity of V. parahaemolyticus at a specific time point. Some V. parahaemolyticus populations might enter a viable but nonculturable (VBNC) stage at low seawater temperature, which may account for the above phenomenon (25).

Our results have significant implications for *Vibrio* disease management. First, recent human activities such as transportation of seafood have overcome the long-standing barriers to the transmission and genetic exchange of V. parahaemolyticus among regions that affect its population structure. Thus, we propose that inspection and quarantining of aquatic animals need to be strengthened to prevent pathogen dispersal in distant locations. Inspection and quarantine of seafood have been established in international trade. However, inspection of aquatic animals and fry is not sufficiently implemented (21). Previous results have suggested that V. parahaemolyticus was introduced to hatchery and nursery facilities through various transmission routes, such as infected nauplii, contaminated feed, or water pipelines contaminated with bacterial organisms (26). Hossain et al. (27) found that the virulent Aeromonas hydrophila isolates from United States-farmed catfish have a recent common ancestor with that found in diseased Asian carp, suggesting potential transmission via aquatic animal transportation. To demonstrate the plausibility of population mixing and genetic exchange within shellfish farms in future work, we will measure the rate of genetic mixing under laboratory conditions, which will greatly improve our understanding of the mutation rate of V. parahaemolyticus.

### Conclusion.

In this study, we introduced the concepts of bacterial communities for the description of intraspecies diversity and dynamics of V. parahaemolyticus. The results here suggested that the transregional transport of live aquatic animals contributed to the population mixing of V. parahaemolyticus. More importantly, these transmission routes allowed genetic exchange among V. parahaemolyticus populations and accelerated bacterial adaptation by transferring ecologically important functions, which provided significant insights into the environmental adaptation of V. parahaemolyticus.

## MATERIALS AND METHODS

### Sampling of seawater in Dalian, Xiamen, and Donggang.

Three shellfish farms were selected for pairwise comparisons of V. parahaemolyticus population dynamics. To evaluate the impacts of an ocean current (starting from the estuary of the Yalu River to Dalian Bay throughout the year) on the population structure of V. parahaemolyticus in Dalian, we selected two shellfish farms on the northeast coast of Dalian (N39.18, E122.11) and Donggang (N39.822, E124.135) that are 160 km apart (Fig. 1A). On 7 June 2018, 1 September 2018, 31 October 2018, 1 February 2019, and 1 May 2019, seawater samples were collected from two farms simultaneously. Each sample, consisting of 2,000 ml of seawater, was collected at 1- and 3-m depths using Niskin bottles and transferred to sterile plastic containers.

To evaluate the impacts of transregional transport of abalones on the local community structure of V. parahaemolyticus, an indoor abalone farm in Xiamen (N24.5167, E118.5967) that received abalones from Dalian on 31 October 2018 was selected. Triplicate 2,000-ml seawater samples were collected at Xiamen at the same five time points as conducted in Dalian and Donggang.

For each sample, 1 liter of seawater was filtered through 0.45-μm-pore-size polycarbonate membranes (Millipore Corporation, Billerica, MA) using a vacuum pump. The filters were immediately placed on thiosulfate-citrate-bile salt-sucrose agar (TCBS) and incubated at 30°C for 24 h. After incubation, all presumptive V. parahaemolyticus colonies were picked and further verified by PCR to detect the presence of a species-specific gene (*toxR* gene) (28).

### MLST of V. parahaemolyticus.

Conventional MLST was performed for all V. parahaemolyticus colonies isolated from the seawater and aquatic animals as previously described (18). Shannon diversity indices and Simpson diversity index values were calculated to assess the population diversity of V. parahaemolyticus in each sample (29).

### Whole-genome sequencing, identification of SNPs, and phylogenetic analyses.

In each sample, one isolate was selected from each predominant ST (>3% population) and used for whole-genome sequencing. High-throughput genome sequencing was carried out on the Illumina platform HiSeq 2500 at Novogene (Beijing, China). The draft genome was assembled *de novo* with SPAdes v3.11.1 (30). The quality of assemblies was assessed by QUAST v4.6.3 (31). SNP calling of the sequenced genome was performed by a previously developed pipeline (32). An in-house Perl script was used to count intergenic, synonymous, and nonsynonymous SNPs (32). The SNP alignment was generated by mapping the reads to the core genome of V. parahaemolyticus defined by Gonzalez-Escalona et al. (33). SNPs located in the recombination regions were removed by ClonalFrameML. RAxML version 8.2.10 was used with the generalized time-reversible model and a Gamma distribution to model site-specific rate variation (with parameters -f a -m GTRGAMMA -p 12345 -x 12345 -# 100) (34). The phylogenetic trees were visualized by Figtree v1.4.4.

### Gene content analysis.

RAST was used to annotate the genomes (35). Antimicrobial resistance genes were identified with ResFinder (36). Each coding sequence initially annotated as integrase, transposase, or recombinase was further annotated by BLASTN against the ISfinder database (37). Mobile gene elements were predicted by VRprofile 2.0 (38).

### Phenotype assays.

To compare the biofilm formation ability between ST722 and ST1808 strains, three independent experiments were performed in 24-well culture plates. Overnight cultures of V. parahaemolyticus were incubated at a concentration of 10^6^ cells ml^−1^ in 1 ml of tryptic soy broth. The microtiter plates were incubated at 25°C for 24 h. V. parahaemolyticus biofilm biomass was determined by 3-(4,5-dimethyl-2-thiazolyl)-2,5-diphenyl-2H-tetrazolium bromide (MTT) staining as described previously (24). As the *cpsA-J* operon (VPA1403 to -1412) in V. parahaemolyticus is essential for exopolysaccharide production, *cpsA* was selected for qPCR to measure the differential expression between ST722 and ST1808 strains as described previously (21). The qPCR assay was performed on three biological and three technical replicates for each sample. Strain ATCC 17082 was used as a calibrator, and fold changes in the gene expression levels relative to it were calculated using the threshold cycle (2^−ΔΔ^*^CT^*) method (39).

### Statistical analysis.

Statistical analyses were conducted using R package version 3.2.3 (40). All statistical tests were considered significant at *P* < 0.05. Differences in V. parahaemolyticus communities from diverse samples were assessed by one-way analysis of similarity (ANOSIM) tests at the ST level. PCoA was conducted to visualize relationships of V. parahaemolyticus composition. Data of phenotype assays were shown as mean ± SD and were compared with one-way analysis of variance (ANOVA), followed by Tukey’s *post hoc* test.

### Data availability.

The raw sequencing data were submitted to GenBank (NCBI) under BioProject no. PRJNA633360.
