# Supplementary figures and images for: Dynamics and Microevolution of Vibrio parahaemolyticus Populations in Shellfish Farms
Source: mSystems. 2021 Jan 12;6(1):e01161-20. doi: 10.1128/mSystems.01161-20 (PMC7901483; doi:10.1128/mSystems.01161-20)

Figure S1

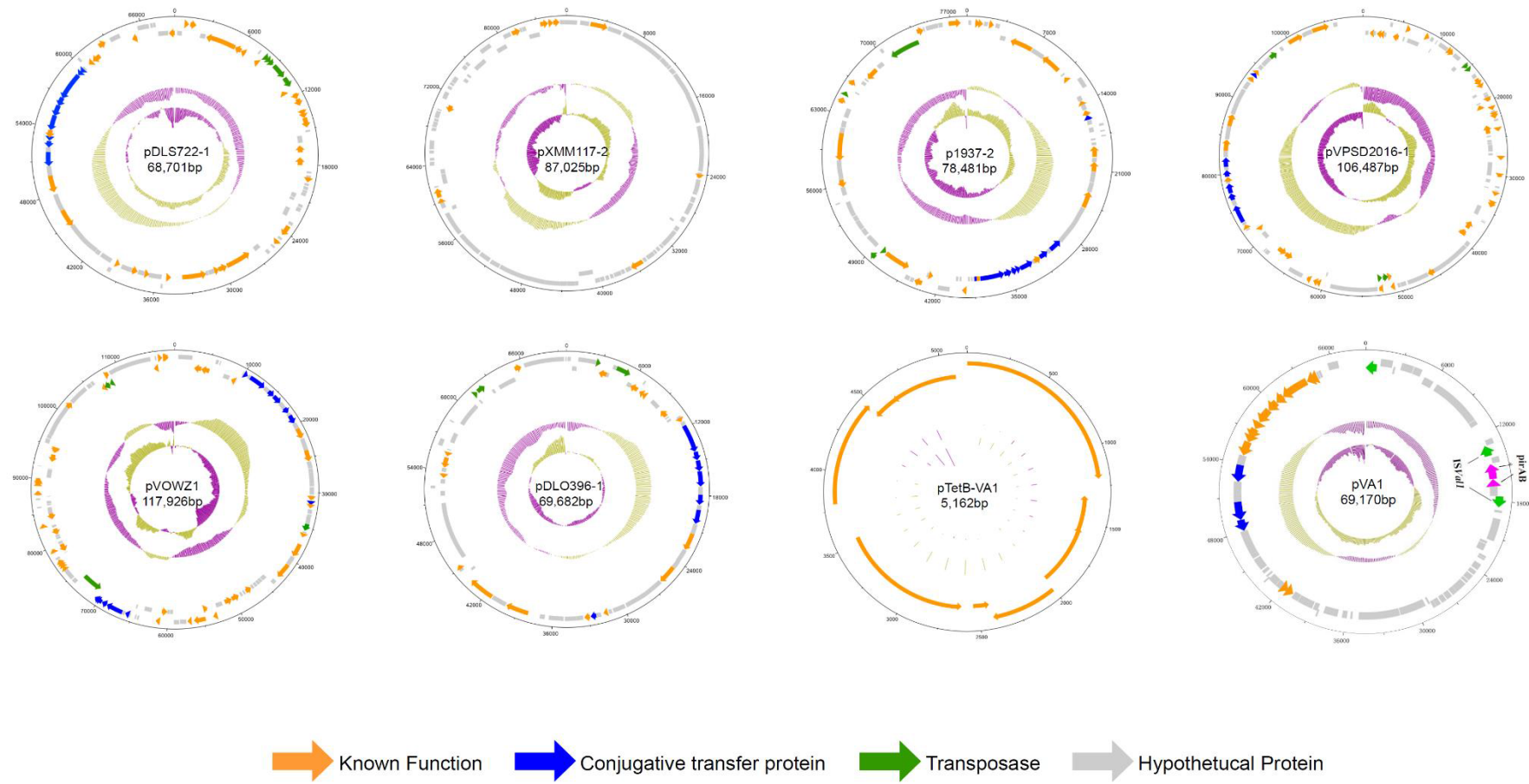

Supplement: FIG S1 [file mSystems.01161-20-sf001.pdf]

Figure S2

A

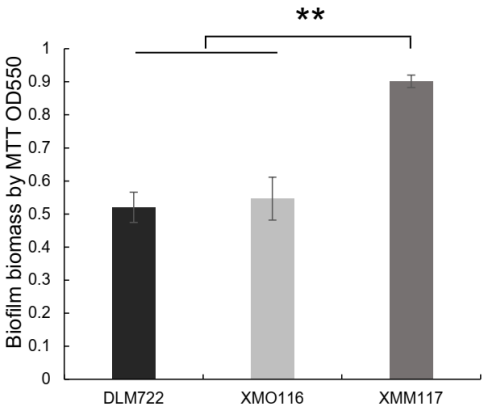

B

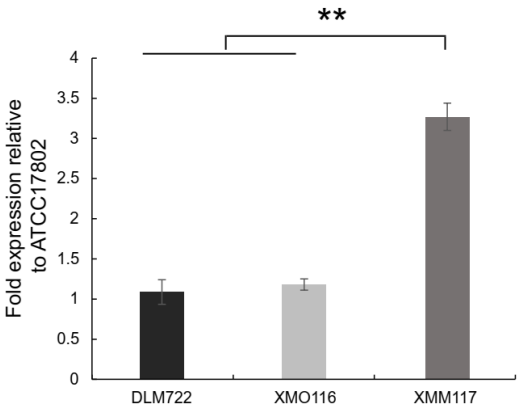

Supplement: FIG S2 [file mSystems.01161-20-sf002.pdf]
